# Activation of HTLV-1 expression in chronically-infected CD4+ T cells: mechanisms and implications for pathogenesis

**DOI:** 10.1186/1742-4690-8-S1-A6

**Published:** 2011-06-06

**Authors:** Alison Swaims, Hsin-Ching Lin, Peter Simon, Celine Granier, Yingyu Zhang, Arthur Roberts, Satish Devadas, Yufang Shi, Arnold B Rabson

**Affiliations:** 1 Child Health Institute of New Jersey, UMDNJ-RWJMS, New Brunswick, New Jersey, 08901, USA; 2 Department of Molecular Genetics, Microbiology, and Immunology UMDNJ-RWJMS, Piscataway, New Jersey, 08854, USA; 3 Cancer Institute of New Jersey UMDNJ-RWJMS, New Brunswick, New Jersey, 08903, USA

## 

Infection with the human T-cell leukemia virus-1 (HTLV-1) is associated with a range of outcomes ranging from asymptomatic infection, to the development of HAM/TSP and adult T cell leukemia/lymphoma. Pathogenesis of these disorders involves complex interactions with the host immune system. Our previous studies showed that phorbol ester (PMA) and T cell receptor (TCR)-mediated activation of chronically infected CD4 T cells increased expression of HTLV-1 gene products [1]. We hypothesized that immune activation of infected T cells may play an important role in disease pathogenesis through induction of Tax expression resulting in increased survival and proliferation. To test this, we employed transgenic mice in which Tax is regulated by the HTLV-1 LTR. These mice develop neurofibromas, but do not express Tax in T cells and do not develop lymphomas [2]. TCR stimulation of transgenic LTR-Tax CD4+ T cells induced Tax expression, early hyper-proliferation, and long-term growth in culture. Survival of these cells was accompanied by transiently increased expression of mcl-1. Long-term surviving cells exhibited a CD4+CD25+CD3- phenotype commonly observed in ATL. Engraftment of immune-activated LTR-Tax CD4+ T-cells into NOD/Shi-scid/IL-2Rγ null mice resulted in a leukemia-like phenotype. Immune activated Tax CD4+ T cells express characteristics of several different CD4+ T cell subtypes, suggesting that HTLV-1 Tax induces changes in the normal pattern of CD4+ subtype specification.

We also investigated mechanisms of HTLV-1 activation following PMA stimulation. Surprisingly, PMA treatment was associated with a rapid, marked stabilization of HTLV-1 tax/rex mRNA. Increased RNA stability represents a novel mechanism for increasing HTLV-1 gene expression in chronically infected cells.
